# Supplementary material for: Comparative genomics provides new insights into the diversity, physiology, and sexuality of the only industrially exploited tremellomycete: Phaffia rhodozyma
Source: BMC Genomics. 2016 Nov 9;17:901. doi: 10.1186/s12864-016-3244-7 (PMC5103461; doi:10.1186/s12864-016-3244-7)
Supplement: Additional file 6: — List of orphan genes with links to PFAM (related to Additional file 1: Table S1). (ZIP 1428 kb) [file 12864_2016_3244_MOESM6_ESM.zip › PFAM_UNIQUE/G05366_P.html]

#                                                                            --- full sequence --- -------------- this domain -------------   hmm coord   ali coord   env coord
  
# target name        accession   tlen query name           accession   qlen   E-value  score  bias   #  of  c-Evalue  i-Evalue  score  bias  from    to  from    to  from    to  acc description of target
  
#------------------- ---------- ----- -------------------- ---------- ----- --------- ------ ----- --- --- --------- --------- ------ ----- ----- ----- ----- ----- ----- ----- ---- ---------------------
  
G05366\_P             -            377 BAF1\_ABF1            PF04684.8    496      0.49    7.8   9.4   1   1    0.0011      0.68    7.4   9.4   305   327   123   145   115   180 0.78 - BAF1 / ABF1 chromatin reorganising factor  
G05366\_P             -            377 SelP\_N               PF04592.9    238       9.2    4.2   9.5   1   1     0.019        17    3.4   9.5   199   220   123   144   109   161 0.53 - Selenoprotein P, N terminal region  
G05366\_P             -            377 Zip                  PF02535.17   317      0.18    9.4   3.0   1   1   0.00082      0.25    9.0   3.0   133   159    66   144    24   170 0.54 - ZIP Zinc transporter
